# Determinants of home delivery among reproductive age women in Bore District, East Guji Zone, Ethiopia: a case–control study

**DOI:** 10.3389/fgwh.2024.1236758

**Published:** 2024-06-07

**Authors:** Beka Teressa, Elsabeth Legesse, Tadesse Nigussie, Berhanu Senbeta Deriba, Ararso Hordofa Guye, Derara Girma, Hiwot Dejene, Leta Adugna, Belete Birhanu, Hana Eshetu, Amanu’el Tadele, Gachena Mideksa

**Affiliations:** ^1^ Department of Public Health, College of Health Sciences, Salale University, Fitche, Ethiopia; ^2^ Department of Nursing, College of Health Sciences, Salale University, Fitche, Ethiopia; ^3^ Department of Public Health, College of Health Sciences, Hawassa University, Hawassa, Ethiopia; ^4^ Department of Public Health, College of Health Sciences, Mizan Tepi University, Mizan Aman, Ethiopia

**Keywords:** pregnancy, home delivery, East Guji, Bore District, Ethiopia

## Abstract

**Introduction:**

Home delivery, which is the process of childbirth at one's residence rather than in a health facility, is a major reason for maternal mortality caused by obstetric complications, such as sepsis, hypertensive disorders, and hemorrhage. Maternal and child mortality remains high in developing countries despite efforts made to reduce these outcomes. This is mainly due to poor utilization of institution-based healthcare services. Moreover, there is a limited number of studies that have addressed the determinants of home delivery in Ethiopia, including the study area. This study aims to identify the determinants of home delivery in Bore District, East Guji Zone, Southern Ethiopia, in 2022.

**Methods:**

A community-based unmatched case–control study was conducted from 18 May to 5 July 2022 among 498 women (249 cases and 249 controls) who gave birth in Bore District. The case group included women who gave birth at home, while the control group included those who gave their last birth at health institutions. A multistage sampling technique was employed to select the study participants. Data were collected using the KoboToolbox digital software and exported to SPSS Version 26.0 for analysis. A multivariable logistic regression analysis was done to declare the statistical significance of the association of the the independent variables and home delivery.

**Results:**

The study included a total of 496 respondents with a mean age of 32.5 (SD = ±5.5) for the case group and 33.7 (SD = ±5.2) for the control group. Among the assessed determinants of home delivery were not attending antenatal care (ANC) visits [adjusted odds ratio (AOR) =  5.6, 95% CI: 2.0–15.16], missing pregnant women's conferences (AOR = 3.2, 95% CI: 1.65–8.32), not receiving health education on pregnancy-related complications (AOR = 2.2, 95% CI: 1.1–4.3), inadequate knowledge of pregnancy-related danger signs (AOR = 6.0, CI: 3.0–11.9), inadequate knowledge about pregnancy-related complications (AOR = 3.0, CI: 1.55–6.13), and unfavorable attitude (AOR = 6.9, 95% CI: 2.16–22.6).

**Conclusion:**

In this study, not attending ANC visits, missing pregnant women's conferences, not receiving health education on pregnancy-related complications, inadequate knowledge of pregnancy-related danger signs, inadequate knowledge about pregnancy-related complications, and unfavorable attitudes were identified as determinants of home delivery. The district health office and other stakeholders should work on strengthening maternal health service delivery through appropriate ANC visits and participation in pregnant women's conferences and improving community awareness about pregnancy at all levels.

## Introduction

Home delivery is a global public health problem in low-income countries. It involves giving birth at one's home rather than at a hospital or health center (1). Giving birth at home is largely unplanned, accidental, and mainly supported by unskilled birth attendants, posing significant risks of morbidity and mortality for both the mother and child (2). Using the home environment as a delivery place is shown to be too unsafe in developing countries (3) and is associated with adverse neonatal and maternal outcomes (4). Institutional delivery is one of the key and proven interventions to reduce maternal death. It ensures a safe birth, reduces both actual and potential complications and maternal death, and increases the survival rate of most mothers and newborns (2, 4). The majority of maternal deaths are due to obstetric complications that could have been prevented with adequate medical care by skilled attendants during and after delivery (5). Lack of professionals’ attendance during home delivery increases the risk of infection by 10%, postpartum hemorrhage (PPH) by 11%, and HIV/AIDS transmission by 6% to relatives or traditional birth attendants who conduct deliveries without protective equipment (6, 7). As a result of these conditions, an estimated 303,000 women die globally every year with almost 99% of deaths occurring in developing countries (8–10).

The worldwide frequency of home births is 28%, with Europe and Central Asia having the lowest prevalence at 5% and the East Asia and the Pacific regions having the highest prevalence at 38% (11, 12). Studies in some countries have reported that home delivery ranges from as low as 22% in Senegal to as high as 65% in Tanzania and 87.7% in Bangladesh (13, 14). The prevalence of this practice in sub-Saharan African countries is 34% on average. Among reproductive-age women in Ethiopia, the prevalence ranges from 13.5% in 2017 to 66.7% in 2020, making Ethiopia one of the countries with the highest rate of home delivery (1, 4). According to the 2019 report of the Ethiopian Demographic and Health Survey (EDHS), 51% of women gave birth at home (15, 16), of which 35% were assisted by TBAs, 13% by relatives or others, and 3% delivered without any type of assistance (15). Another study conducted in the Oromia region of Ethiopia showed that approximately 59.1% of women gave birth at home, which was higher than that at the national level (15).

A community-based cross-sectional study conducted in Shashamanne town, Ethiopia, revealed a home delivery rate in the town of 68%, due to the absence of labor pain (75.5%), lack of adequate delivery services from health professionals (5.7%), and lack of knowledge about the importance of delivery in health institutions (15.1%) (17).

Studies conducted so far identified residence, educational level of the mother, knowledge of obstetric complications, and gestational age at first antenatal care (ANC) as the main individual-level factors associated with home delivery (1, 5, 16, 17, 18). Few studies also revealed the following community-level factors that determine decisions for home delivery: women living in urban settings, who are literate and influenced by their husbands and relatives, and who have had prolonged labor are more likely to deliver in health facilities (1, 5, 16, 19).

To address the delays of pregnant women in their decisions to seek and receive care from health facilities and their consequences, the Federal Ministry of Health of Ethiopia has shown a strong commitment by promoting human resource development and bringing maternal healthcare services closer to women. The ministry has so far launched an initiative with the slogan “No Woman Should Die While Giving Life” and the general motto “Home Delivery Free Kebeles” (HDF) (15, 16). The Women's Development Army (WDA) also plays a critical role in minimizing the magnitude of home delivery at the local level by identifying pregnant women in the community (20).

Despite the efforts made by the government and other stakeholders to reduce the problems and subsequent consequences of home delivery, studies conducted in different parts of the country showed that most Ethiopian women are giving birth at home and are experiencing problems associated with it (10, 15, 16, 18, 21). With these problems in mind, this study aims to identify the determinants of home delivery in the study area and enable a deeper understanding of the study participants' experiences of issues associated with home delivery.

### Conceptual framework

**Figure 1:**
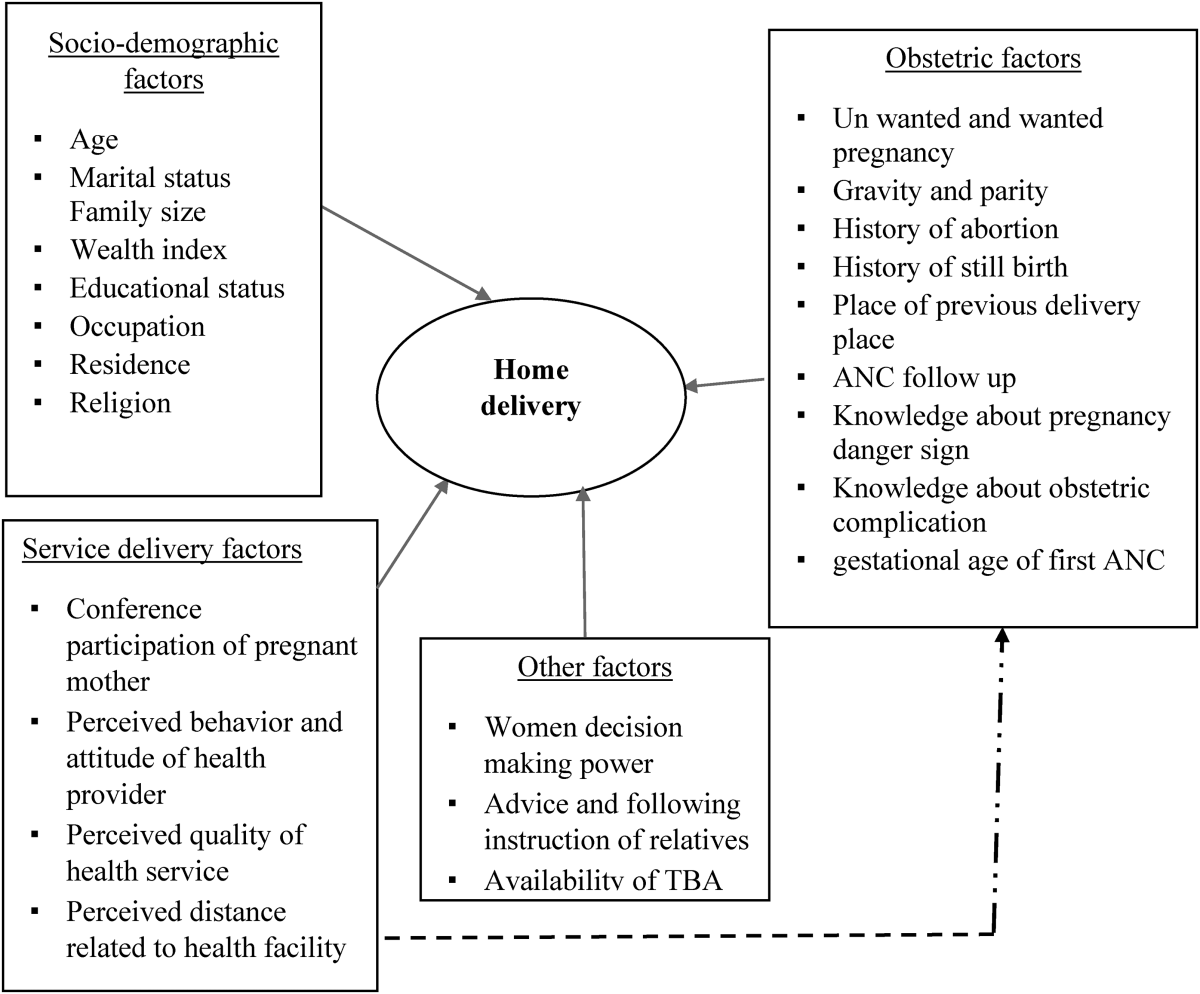
Conceptual framework for the factors associated with home delivery in Bore District, East Guji Zone, Southern Ethiopia, in 2022.

The conceptual framework of this study was adapted from related published literature grouped into sociodemographic, obstetric, service provider, and health facility-related factors (16, 20, 21) (Figure 1).

## Methods and materials

### Study design, period, and area

A community-based, unmatched case–control study was conducted in Bore District from 18 May to 5 July 2022. The district is one of the areas found in the East Guji Zone, Oromia Region, and is located 450 km away from Addis Ababa. The district has 33 kebeles (3 urban and 30 rural kebeles). There are six public health centers, one primary hospital, 33 health posts, and 10 private clinics in the district that provide comprehensive health services, including delivery service, which is free of charge. The total population of the district is approximately 174,941 according to the basic data obtained from the district health office in 2022 (22).

According to the 2021/2022 report of the district health office, the expected number of deliveries is 6,070, of which 2,792 occurred at health facilities. The report from the previous 6 months, based on the data from the district’s health post-family folder, indicated that there were 1,298 home deliveries and 1,690 institutional deliveries (Bore District Health Office, 2022)^1^.

### Study population

All women who gave birth in Bore District 6 months prior to the study were the source population. All randomly selected women who gave birth in the same district 6 months prior to the study were the study population. Regarding the eligibility criteria, all women who gave birth within 6 months prior to the study and lived in the district for at least 6 months were included in the study.

### Sample size determination

The sample size required was calculated using a two-population proportion formula and the EPI INFO version 7.0 statistical software, with an assumption of 95% CI (*Z*α/2 = 1.96), power of 80% (*Z* = 0.84), and case-to-control ratio of 1:1, and by taking P1 as the proportion of cases exposed and P2 as the proportion of controls exposed. Residence in rural mothers was taken as an exposure variable (23) (Table 1).

**Table 1:** Sample size determination for the factors associated with home delivery in Bore District, East Guji Zone, Ethiopia, in 2022.

S. no.	Variables	CI	Power	P1	P2	Odds ratio	Sample size			References
							Number of cases	Number of controls	Total	
1	Unable to read and write (education status)	95%	80%	32.2	14.3	2.85	97	97	196	(24)
2	Rural resident	**95%**	**80%**	**20**.**0**	**6**.**48**	**3**.**6**	**113**	**113**	**226**	(23)
3	ANC (less than three visits)	95%	80%	21.9	4.47	6	69	69	138	(24)
4	Husbands decided place of delivery	95%	80%	68.0	22.8	7.2	23	23	46	(5)
5	No awareness of pregnancy-related danger signs	95%	80%	60.7	31.9	3.30	53	53	106	(21)
6	Time to health facility >1 h	95%	80%	18.9	4.92	4.5	97	97	194	(24)
7	Negative attitude	95%	80%	37.1	13.6	3.74	61	61	122	(24)
8	Poor knowledge of obstetric complication	95%	80%	90.4	54.1	7.95	28	28	56	(19)
9	Gestational age >16 weeks	95%	80%	55.9	21.6	4.6	37	37	74	(5)
10	Not involved in WDA	95%	80%	45.5	20.2.	3.3	61	61	122	(5)
11	Maternal age <25 years	95%	80%	40.9	19	2.95	77	77	154	(21)

Bold values indicate significant variables considered to calculate sample size from previously published literature.

The calculated sample size with the maximum sample for this study was 226. Considering the design effect of 2% and 10% non-response rates, the final sample size was found to be 498, with a total of 249 cases and 249 controls.

### Sampling procedure

A multistage sampling technique was used to select study participants from the selected kebeles in the district. Initially, the kebeles found in the district were stratified into rural and urban kebeles. Ten rural and 2 urban kebeles were chosen randomly (using a lottery method) from the 33 kebeles found in the district (3 urban and 30 rural kebeles). Regarding the selection of participants, first, the list of 1,097 eligible women who gave birth in the selected kebeles was taken from family folders found in their respective health posts and used as a sampling frame. Next, all eligible cases and controls were separately identified with their full address from the family folder of nearby health posts for each selected kebele before the actual data collection. A family folder is a registry containing the profiles of all the family members in the kebele. The list of eligible mothers that was obtained from the health posts’ family folder was cross-checked with the delivery records in the health posts to ensure that no eligible mother was left out of the sampling frame. Proportional allocation to size was then made to determine the required sample size from each kebele. Finally, a simple random sampling technique was used to select the required 249 cases and 249 controls from each kebele using the household listed as a sampling frame. Local guides were used to reach the mothers. A minimum of three times of revisits was arranged for the eligible mothers who were not present at the time of data collection. The lottery method was used to select one woman in case there were two or more women in an eligible household.

### Data collection tool and procedures

Data were collected through face-to-face interviews using interviewer-administered structured questionnaires that were developed from a review of available literature (9, 24, 25). The questionnaire had four parts: sociodemographic characteristics, maternal and obstetric characteristics, facility and service provider characteristics, and cultural beliefs of the respondents. Cronbach's *α* coefficients were computed to test the internal consistency/reliability of attitude toward delivery services. This was measured using six-point Likert scale-based items, and Cronbach's *α* was calculated to be 0.84. Six health officers who were recruited from the district health office conducted data collection under the supervision of two public health workers with a master’s degree who also came from the district health office.

### Study variables

#### Dependent variable

Place of delivery (home delivery).

#### Independent variables

Sociodemographic characteristics: age, marital status, type of marriage, family size, wealth index, educational status, occupation, religion, and place of residence.

Obstetric characteristics: age at first birth, ANC visit, unwanted pregnancy, unplanned pregnancy, knowledge of pregnancy-related danger signs, knowledge of obstetric complications, gestational age at first ANC, gravidity, parity, history of abortion, history of stillbirth, and place of the previous delivery.

Service provider- and health facility-related factors: health information on pregnancy-related conditions, perceived behavior and attitude of health provider, perceived quality of health service, counseling to deliver at a health facility, and perceived distance to a health facility.

Other factors: women's decision-making power, adherence to advice and instructions from relatives, and availability of traditional birth attendants.

### Quality assurance

The questionnaire was prepared in English and translated to Afan Oromo by a senior language translator for better understanding and then translated back to English by an independent person to ensure consistency. The data collectors and supervisors received a 2-day training covering the objective of the study, contents of the questionnaire, confidentiality protocols, rights of the respondents, and data collection approach. A pretest was conducted on 5% of the samples at the Haro kebele, a neighboring kebele to the study district. Following data collection, the principal investigator and supervisors checked the completeness, accuracy, clarity, and consistency of the collected data before downloading and exporting them from the KoboToolbox to Excel. Data in Excel were then converted to SPSS to check for possible outliers, missing values, fulfillment of assumptions, and feasibility for analysis.

### Data processing and analysis

The data were collected using the KoboToolbox software; downloaded from there; coded, cleaned, and corrected as needed on Excel; and then exported to SPSS Version 26 for data cleaning and analysis. The exported data were checked for outliers, missing values, and assumptions. The descriptive variables were explained using cross-tabulations, and frequencies were generated for different variables as needed. A bivariable analysis was carried out to identify the candidate variables for multivariable logistic regression. The independent variables with a *p*-value of <0.25 in the bivariate analysis were then used for further analysis through multivariable logistic regression (to control the confounding effects). The backward model selection method was employed to identify the variables remaining for the final model. Multicollinearity was checked with a standard error (SE) of 2 as the cutoff point. All variables in this study were found to be less than 2 SE. Model fitness was checked with the Hosmer and Lemeshow model goodness-of-fit test at a *p*-value of > 0.05. Finally, an AOR with a 95% CI at a *p*-value of <0.05 was used to declare the statistical significance of the association between the dependent variables (home delivery) and selected independent variables.

### Measurements and operational definitions

The case group comprised women who had their recent births at home, while the control group comprised women who had their last births at health institutions (16).

Knowledge of participants on the danger signs during pregnancy was measured by using 10 multiple-choice questions about danger signs. The total score for knowledge was dichotomized into inadequate and adequate knowledge by the mean score cutoff point. Inadequate knowledge is defined as a score less than or equal to the mean score for knowledge questions, while adequate knowledge is defined as a score greater than the mean score for knowledge questions (16, 23).

Knowledge of participants on general obstetric complications was measured by using eight multiple-choice questions about obstetric complications. The total score for knowledge was dichotomized into inadequate and adequate knowledge by the mean score cutoff point. Inadequate knowledge is defined as a score less or equal to the mean score for knowledge questions, while adequate knowledge is defined as a score greater than the mean score for knowledge questions (16, 23).

#### Proximity to a health facility

A participant was considered close to a health facility if she traveled less than or equal to an hour on foot to reach the health facility and considered far from a health facility if she traveled greater than an hour on foot to reach the health facility (21).

#### Maternal conference

Maternal conference is the practice in which pregnant women form a monthly gathering with one another and with health professionals to discuss their health and well-being.

#### Attitude toward institutional delivery service

Six-point Likert scale questions were used to assess the attitude of the participants. The variable for attitude was dichotomized into unfavorable and favorable attitudes, based on the mean score cutoff point. An unfavorable attitude is defined as a score less or equal to the mean score for attitude questions, while a favorable attitude is defined as a score greater than the mean score for attitude questions (16, 23).

#### Wealth index

The wealth index was assessed by using the Equity Tool (asset variables: access to electricity and ownership of electric appliances such as refrigerators, televisions, and radios) and analyzed using the principal component analysis. Before conducting the principal component analysis, the value of each wealth variable was categorized as 0 = no and 1 = yes. After conducting the analysis, the variables were categorized into the first, second, third, fourth, and fifth quintile groups and then transformed into three categories representing the lower, middle, and higher wealth statuses.

## Results

### Sociodemographic characteristics of the respondents

**Figure 2:**
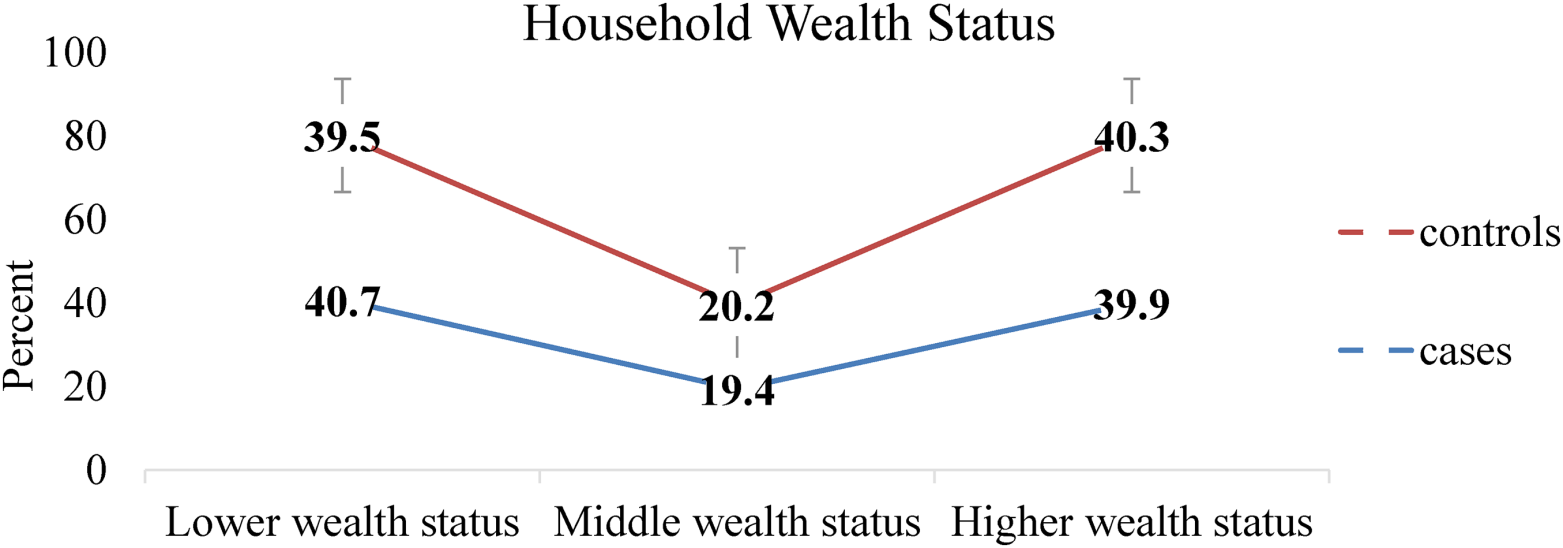
Wealth status of households with women who gave birth in Bore District, East Guji Zone, Southern Ethiopia, in 2022.

A total of 496 respondents (248 cases and 248 controls) were involved in this study, resulting in a response rate of 99.6%. The mean age of the study participants was 32.5 (SD = ±5.5) for the case group and 33.7 (SD = ±5.2) for the control group. The majority of the participants in the case group (62.9%) and the control group (58.5%) lived in rural areas. Regarding educational status, 44% of mothers and 18.1% of husbands in the case group and 21% of mothers and 5.6% of husbands in the control group were unable to write and read (Table 2). The wealth index of the households revealed that 39.5% of the control group and 40.7% of the case group had a lower wealth status (Figure 2).

**Table 2:** Sociodemographic characteristics of the study participants on home delivery in Bore District, East Guji, Ethiopia, in 2022.

Variables	Cases (*n* = 248)	Controls (*n* = 248)
	No. (%)	No. (%)
Age (in years)		
15–19	24 (9.6)	15 (6.0)
20–24	76 (30.6)	15 (6.0)
25–29	88 (35.4)	55 (22.1)
30–34	32 (12.9)	85 (34.2)
35–39	28 (11.2)	78 (31.4)
Residence		
Rural	156 (62.9)	145 (58.5)
Urban	92 (37.1)	103 (41.5)
Educational status		
Unable to write and read	109 (44)	52 (21)
Primary school	85 (34.3)	73 (29.4)
High school and preparatory	39 (15.7)	87 (35.1)
Diploma and above	15 (6)	36 (41.5)
Marital status		
Single	16 (6.5)	6 (2.4)
Married	211 (85.1)	216 (87.1)
Widowed	12 (4.8)	21 (8.5)
Divorced	9 (3.6)	5 (2)
Type of marriage		
Monogamy	123 (49.6)	89 (35.9)
Polygamy	125 (50.4)	159 (64.1)
Occupation		
Farmer	32 (12.9)	32 (12.9)
Housewife	146 (58.9)	135 (54.4)
Merchant	54 (21.8)	55 (22.2)
Government employee	16 (6.5)	26 (10.5)
Religion		
Orthodox	68 (27.4)	63 (25.4)
Protestant	146 (58.9)	149 (60.1)
Muslim	34 (13.7)	36 (14.5)
Ethnicity		
Oromo	202 (81.5)	190 (76.6)
Amhara	37 (14.9)	43 (17.3)
Others*	9 (3.6)	15 (6)

* Tigre, guraghe, and silte.

### Obstetric characteristics of the respondents

A total of 187 (75.4%) cases and 181 (73.8%) controls had a parity of 2–5. Accordingly, 156 (62.9%) cases and 89 (35.9%) controls had no ANC visit in their last pregnancy. The last pregnancy of 175 (70.6%) cases and 220 (88.7%) controls was planned. Only 78 (31.5%) cases and 232 (93.5%) controls reported that giving birth at home is risky for both the fetus and the mother (Table 3).

**Table 3:** Obstetric-related characteristics of women who gave birth in the last 6 months in Bore District, East Guji Zone, Southern Ethiopia, in 2022.

Variables	Cases (*n* = 248)	Controls (*n* = 248)
	No. (%)	No. (%)
Gravida		
Primigravida	15 (6.0)	24 (9.7)
Multigravida	233 (94.0)	224 (90.3)
ANC visits		
No ANC visit	156 (62.9)	89 (35.9)
One–three visits	67 (27.0)	116 (46.8)
Four and above visits	25 (10.1)	43 (17.3)
Place of ANC visit		
Health center	24 (26.1)	104 (65.4)
Health post	64 (69.6)	36 (22.6)
Hospital	4 (4.3)	19 (11.9)
Gestational age at first ANC visit		
Less than or equal to 16 weeks	10 (11)	30 (18.9)
Greater than 16 weeks	81 (89)	129 (81.1)
Last pregnancy intended		
Yes	181 (73)	219 (88.3)
No	67 (27)	29 (11.7)
History of abortion		
Yes	16 (6.5)	28 (11.3)
No	232 (93.5)	220 (88.7)
History of stillbirth		
Yes	5 (20)	17 (6.9)
No	243 (98)	231 (93.1)
Level of knowledge of pregnancy-related danger signs		
Inadequate knowledge	134 (72)	45 (18.8)
Adequate knowledge	52 (28)	194 (81.2
Level of knowledge on obstetric complication		
Inadequate knowledge	100 (66.2)	46 (19.7)
Adequate knowledge	51 (33.8)	188 (80.3)
Importance of delivery at a health facility		
Yes	150 (60.5)	246 (99.2)
No	98 (39.5)	2 (0.8)

### Health facility- and service provider-related factors

The number of study participants who did not receive counseling about the place of delivery was 125 (50.4%) for the case group and only 12 (4.8%) for the control group. A total of 239 (96.4%) cases and 190 (76.6%) controls had an unfavorable attitude toward health facility delivery service (Table 4).

**Table 4:** Healthcare provider-related characteristics of the study participants in Bore District, East Guji Zone, Southern Ethiopia, in 2022.

Variable	Cases (*n* = 248)	Controls (*n* = 248)
	No. (%)	No. (%)
Ever got counseling about the place of delivery		
No	125 (50.4)	12 (4.8)
Yes	123 (49.6)	236 (95.2)
Health education on pregnancy-related complications		
No	199 (80.2)	168 (43.5)
Yes	49 (19.8)	140 (56.5)
Maternal conference participation		
No	206 (83.0)	199 (40.0)
Yes	42 (17.0)	49 (60.0)
Proximity to a health facility		
Closest to the health facility	49 (19.8)	36 (14.5)
Far from health facility	199 (80.2)	212 (85.5)
Access to ambulance service		
No	147 (59.3)	111 (44.8)
Yes	101 (40.7	137 (55.2)
Attitude toward the facility delivery service		
Unfavorable attitude	239 (96.4)	190 (76.6)
Favorable attitude	9 (3.6)	58 (23.4)

### Availability of traditional birth attendants and decision-making power regarding the place of delivery

**Figure 3:**
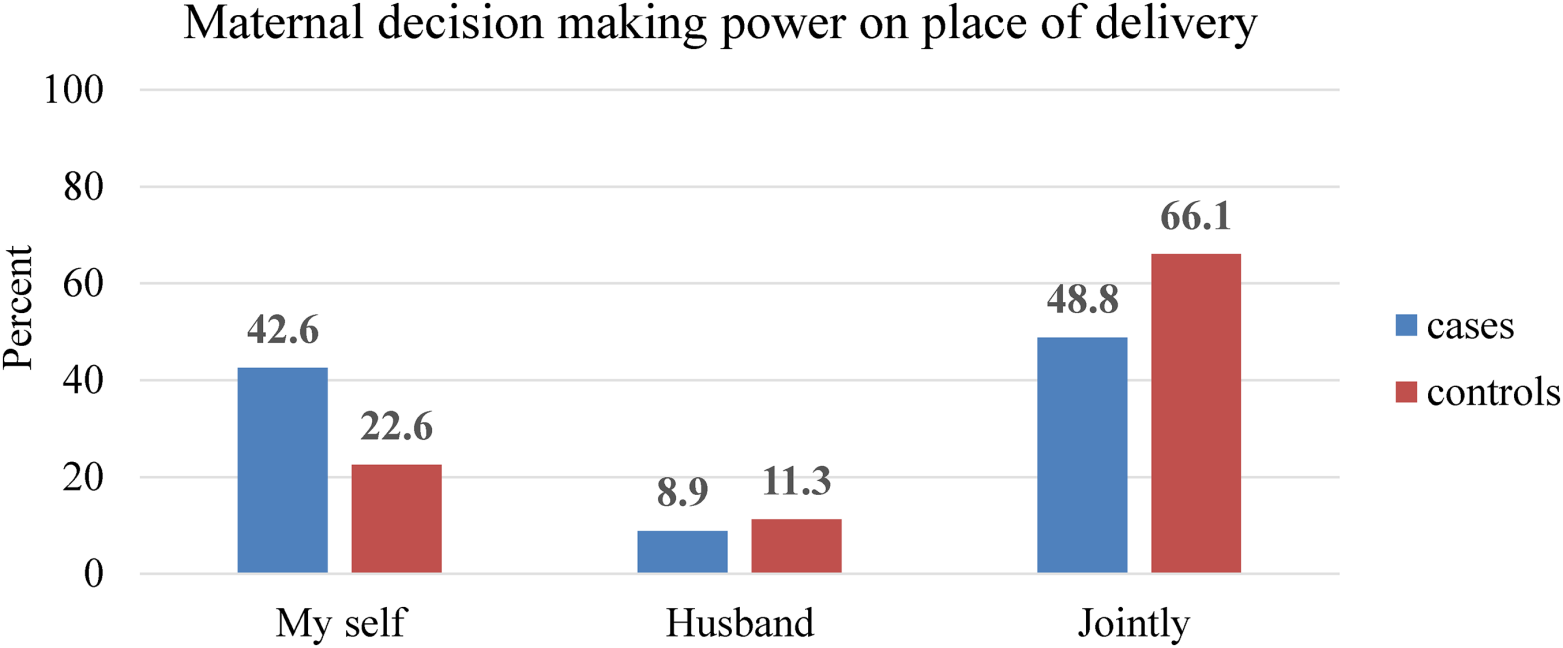
Maternal decision-making level on the delivery site in the last pregnancy in Bore District, East Guji Zone, Southern Ethiopia, in 2022.

A total of 187 (75.4%) cases and 178 (71.8%) controls reported the presence of traditional birth attendants in their locality. Regarding the decision-making of the delivery place, only 105 (42.6%) cases and 56 (22.6%) controls indicated that the decision was independently made by mothers (Figure 3).

### Determinants of home delivery

The study showed that women who had no ANC visit had 5.6-fold higher odds of giving birth at home compared to those who had four or more ANC visits (AOR = 5.6, 95% CI: 2.06–15.16). Women who had inadequate knowledge of pregnancy-related danger signs were six times more likely to give birth at home compared to their counterparts (AOR = 6.0, CI: 3.0–11.9). The study also revealed that women who had inadequate knowledge of obstetric complications had threefold higher odds of giving birth at home compared to those who had adequate knowledge of the issue (AOR = 3.0, CI: 1.55–6.13). Women who did not participate in pregnant women’s conferences had 3.2 times higher odds of giving birth at home compared to those who participated in the conference thrice and higher (AOR = 3.2, 95% CI: 1.65–8.32).

Women who did not receive health education on pregnancy and pregnancy-related complications were 2.2 times more likely to give birth at home compared to their counterparts (AOR = 2.2, 95% CI: 1.1–4.3). Women who had an unfavorable attitude toward health facility delivery services had seven times higher odds of giving birth at home compared to those who had a favorable attitude toward health facility delivery services (AOR = 6.9, 95% CI: 2.16–22.6) (Table 5).

**Table 5:** Determinants of home delivery in Bore District, East Guji Zone, Southern Ethiopia, in 2022.

Variables	Cases No. (%)	Controls No. (%)	COR (95% CI)	AOR (95% CI)	*p*-value
ANC visits					
No visit	156 (62.9)	89 (35.9)	3 (1.7–5.2)	5.6 (2.06–15.16)	0.001*
One–three visits	67 (27)	116 (46.8)	1 (0.5–1.7)	1.1 (0.4–3.03)	0.287
≥4 visits	25 (10.1)	43 (17.3)	1	1	
Knowledge of pregnancy-related danger signs					
Inadequate	134 (72)	45 (18.8)	11 (7.0–17.5)	6 (3.0–11.9)	0.001*
Adequate	52 (28)	194 (81.2)	1	1	
Knowledge of obstetric complication					
Inadequate	100 (66.2)	46 (19.7)	8 (5.02–12.7)	3 (1.55–6.13)	0.001*
Adequate	51 (33.8)	188 (80.3)	1	1	
Pregnant women’s conference participation					
No	206 (83.0)	199 (40.0)	1.2 (1.08–3.8)	3.2 (1.6–8.3)	0.024*
Yes	42 (17.0)	49 (60.0)	1	1	
Health education on pregnancy-related complications					
No	199 (80.2)	108 (43.5)	5.2 (3.5–7.8)	2.2 (1.1–4.3)	0.020*
Yes	49 (19.8)	140 (56.5)	1	1	
Attitude toward the delivery service					
Unfavorable, favorable	239 (96.4)	190 (76.6)	8 (3.91–16.8)	6.9 (2.16–22.6)	0.001*
	9 (3.6)	58 (23.4)	1	1	

* Significant at a *p*-value of <0.05.

## Discussion

This study aimed to assess the determinants of home delivery among women who gave birth in Bore District, Ethiopia. The study found that mothers' lack of ANC visits, mother’s lack of participation in pregnant women's conferences, unfavorable attitude toward institutional delivery, inadequate knowledge of pregnancy-related danger signs, mothers' lack of health education on pregnancy and pregnancy-related problems, and inadequate knowledge of obstetric complications are the determinants of home delivery.

The study indicated that women who had inadequate knowledge of pregnancy-related danger signs had increased odds of giving birth at home compared to their counterparts. This result is consistent with the findings of studies conducted in Northern Ethiopia and Bahirdar (16, 23). The reason for this might be because women with inadequate knowledge of pregnancy-related danger signs do not know the consequences of home delivery and, therefore, may not hesitate to do so.

Women who had an unfavorable attitude toward institutional delivery service were more likely to give birth at home compared to those who had a positive attitude toward such service. This finding agrees with the results of studies conducted in Zala woreda, Southern Ethiopia, and Eritrea (24, 26). The unfavorable attitude of women toward such service might result from missing expected service elements like lack of privacy to one's own body in every visit to the health facility or actual or assumed ill treatment of the mothers by health professionals. Such an attitude may promote risk/consequences associated with home delivery. The current study showed that the odds of home delivery were considerably higher among women with inadequate knowledge of obstetric complications. These findings are consistent with the results of previous studies in the Afar region, Ethiopia, and other countries such as Nepal (21, 27). The increased understanding that mothers have about obstetric complications may make them fear these problems after giving birth; therefore, they prefer institutional delivery being assisted by skilled professionals.

Being unable to participate in pregnant women's conferences was significantly associated with home delivery in the current study. Women who were unable to participate in the conferences were more likely to give birth at home compared to conference participants. This finding is supported by a study done in Northwest Ethiopia (28). Women who were not involved in the pregnant women's conferences lack information about the benefits of giving birth at a health facility and the complications of home delivery and therefore intend to deliver at home.

Women who had no ANC visit were more likely to give birth at home when compared to those who had four or more ANC visits. This finding is in line with the results of studies conducted in the Tanqua Abergele District in Northern Ethiopia, East Wollega, Zala woreda in Southern Ethiopia, other countries such as Nepal, and Eretria (16, 24, 26, 27, 29). The possible reason may be that women who make fewer visits would be less likely to obtain adequate information and counseling about the advantages of health facility delivery and will therefore favor home delivery over facility visits. Nonetheless, a study done in Nigeria argues that having an ANC visit was associated with an increased risk of home delivery as women who are told their pregnancy is fine may feel encouraged to deliver at home (30).

Exposure to health education on pregnancy and pregnancy-related problems was another predictor of home delivery in this study. Women who lacked health education on pregnancy and pregnancy-related problems had considerably higher odds of giving birth at home compared to their counterparts. This finding agrees with the results of studies conducted in Shashamanne and Hamer, Ethiopia (5, 31). The health education given may enable women to acquire knowledge on pregnancy and obstetric complications, consequences of delivering at home, and care the mother and child can obtain from delivering at a health institution and may therefore make them develop an interest in delivering at such a place.

### Strength and limitation of the study

This study used a community-based case–control study design that could give a strong prediction of home deliveries. As a limitation, the study may be susceptible to recall and social desirability bias since the method involved interviewer-administered questionnaires in data collection. Although multistage sampling is utilized to sample the study populations and gather data, the analysis did not take this attribute into account because data on the variables used for forming clusters were not collected. Finally, there were missing data for some variables.

### Conclusion and recommendation

The lack of ANC visits, inadequate knowledge of pregnancy-related danger signs and obstetric complications, lack of health education on pregnancy and pregnancy-related complications, unfavorable attitude toward facility delivery services, and lack of PWC attendance were the identified determinants of home delivery.

The district health office should work to improve institutional delivery through appropriate ANC visits, strengthening pregnant women’s conferences, and improving community awareness about pregnancy at all levels. Health professionals should give due attention to pregnancy-related danger signs and complications and husband involvement in pregnant women’s conferences. All health facilities in the district should arrange PWC every 2 weeks in each kebele and monthly in health centers in collaboration with HEWs and midwives.

## Data Availability

The raw data supporting the conclusions of this article can be made available by the authors, without undue reservation.

## Footnotes

^1^Source: Bore District Health Office Basic Profile Data, unpublished.

## References

[1] Teferi HM San Sebastian M Baroudi M. Factors associated with home delivery preference among pregnant women in Ethiopia: a cross-sectional study. Glob Health Action. (2022) 15(1):1–9. 10.1080/16549716.2022.2080934 PMC9310790 35867544

[2] Health W. Maternal Health. In: guidelines approved by the WHO review committee (2017). p. 36.

[3] Prata N Bell S Quaiyum MA. Modeling maternal mortality in Bangladesh: the role of misoprostol in postpartum hemorrhage prevention. BMC Pregnancy Childbirth. (2014) 14:1–10. 10.1186/1471-2393-14-78 PMC3932142 24555848

[4] Ayele GS Melku AT. Utilization of skilled birth attendant at birth and associated factors among women who gave birth in the last 24 months preceding the survey in Gura Dhamole Woreda, Bale Zone, Southeast Ethiopia. BMC Public Health. (2019) 19:1–14. 10.1186/s12889-019-7818-6 31711460 PMC6849185

[5] Markos E. Determinants of home delivery among women in rural pastoralist community of Hamar District, Southern Ethiopia: a case–control study. Risk Manag Healthc Policy. (2020) 13:2159–67. 10.2147/RMHP.S268977 PMC7575064 33116994

[6] FDRE M. Federal Democratic Republic of Ethiopia Ministry of Health Sector Development Program IV October 2010 contents (2014). (October 2010).

[7] FMOH. Ethiopian Health Sector Transformation Plan. 2015/16–2019/20. Fed Democr Repub Ethiop Minist Heal (2015). 20(May): p. 50.

[8] Delibo D Damena M Gobena T Balcha B. Status of home delivery and its associated factors among women who gave birth within the last 12 months in East Badawacho. BioMed Res Int. (2020):1–8. 10.1155/2020/4916421 32923481 PMC7453228

[9] Moindi RO Ngari MM Nyambati VCS Mbakaya C. Why mothers still deliver at home: understanding factors associated with home deliveries and cultural practices in rural coastal Kenya, a cross-section study global health. BMC Public Health. (2016) 16(1):1–8. 10.1186/s12889-016-2780-z 26842657 PMC4738797

[10] Nigusie A Azale T Yitayal M. Institutional delivery service utilization and associated factors in Ethiopia: a systematic review and meta-analysis. BMC Pregnancy Childbirth. (2020) 5:1–25. 10.1186/s12884-020-03032-5 PMC7296650 32539698

[11] Id SH Gilano G Simegn AE Tariku B Id S. Spatial variation and determinant of home delivery in Ethiopia: spatial and mixed effect multilevel analysis based on the Ethiopian Mini Demographic and Health Survey 2019. PLoS One. (2022) 17:1–16. 10.1371/journal.pone.0264824 PMC8916675 35275944

[12] Tariku M Enyew DB. Home delivery among pregnant women with ANC follow-up in Ethiopia; evidence from the Ethiopia Mini Demographic and Health Survey. Front Public Heal. (2022) 16:1–8. 10.3389/fgwh.2024.1236758 PMC9709139 36466499

[13] Wahed T Moran AC Iqbal M. The perspectives of clients and unqualified allopathic practitioners on the management of delivery care in urban slums, Dhaka, Bangladesh—a mixed method study. BMC Pregnancy Childbirth. (2010) 10:1–9. 10.1186/1471-2393-10-50 20822521 PMC2940791

[14] Mrisho M Schellenberg JA Mushi AK Obrist B Mshinda H Tanner M. Factors affecting home delivery in rural Tanzania. Trop Med Int Health. (2007) 12(7):862–72. 10.1111/j.1365-3156.2007.01855.x 17596254

[15] Ethiopian Public Health Institute (EPHI) [Ethiopia] and ICF. Ethiopia Mini Demographic and Health Survey 2019: Key Indicators. Rockville, Maryland, USA: EPHI and ICF (2019).

[16] Tsegay R Aregay A Kidanu K Alemayehu M Yohannes G. Determinant factors of home delivery among women in Northern Ethiopia: a case control study. BMC Public Health. (2017) 17(1):1–8. 10.1186/s12889-017-4159-1 28372540 PMC5379537

[17] Mitiku AA Dimore AL Mogas SB. Determinants of home delivery among mothers in Abobo District, Gambella Region, Ethiopia: a case control study. Int J Reprod Med. (2020) 2020:1–7. 10.1155/2020/8856576 PMC7787860 33490230

[18] Manote M Gebremedhin T. Determinants of postnatal care non-utilization among women in Demba Gofa rural district, Southern Ethiopia: a community-based unmatched case–control study. BMC Pregnancy Childbirth. (2020) 9:1–10. 10.1186/s12884-020-03244-9 PMC7501668 32948140

[19] Kebede ZT. Determinants of health facility delivery in Northwest Ethiopia: a community-based case–control study. Int J Gen Med. (2021) 14:993–1001. 10.2147/IJGM.S300178 PMC8001102 33790628

[20] Bukola L Dorothy T Joseph S. Determinants of choice of birth place among women in rural communities of Southwestern Nigeria. Int J Africa Nurs Sci. (2020) 13(April):29. 10.1016/j.ijans.2020.100244

[21] Abdella M Abraha A Gebre A Surender Reddy P. Magnitude and associated factors for home delivery among women who gave birth in last 12 months in Ayssaita, Afar, Ethiopia—2016. A community-based cross-sectional study. Glob J Fertil Res. (2017) 2(1):30–9. 10.17352/gjfr.000009

[22] Adugna A. Demography and health in Oromia (2021). p. 27.

[23] Abebe F Berhane Y Girma B. Factors associated with home delivery in Bahirdar, Ethiopia: a case control study. BMC Res Note. (2012) 5:1–6. 10.1186/1756-0500-5-653 PMC3554461 23176369

[24] Kucho B Mekonnen N. Delivery at home and associated factors among women in child bearing age, who gave birth in the preceding two years in Zala Woreda, southern Ethiopia. Acad J. (2017) 9(June):177–88. 10.5897/JPHE2017.0921

[25] Duko B Toma A Abraham Y. Alcohol use disorder and associated factors among individuals living with HIV in Hawassa City, Ethiopia: a facility based cross-sectional study. Subst Abus Treat Prev Policy. (2019) 14(1):1–6. 10.1186/s13011-018-0189-7 PMC6528325 31109353

[26] Kifle MM Kesete HF Gaim HT Angosom GS Araya MB. Health facility or home delivery? Factors influencing the choice of delivery place among mothers living in rural communities of Eritrea. J Health Popul Nutr. (2018) 37(1):22. 10.1186/s41043-018-0153-1 30348219 PMC6196428

[27] Tuladhar H. Determinants of home delivery in a semi urban setting of Nepal. Nepal J Obstet Gynecol. (2009) 4(1):30–7. 10.3126/njog.v4i1.3329

[28] Asresie MB. Effect of attending pregnant women’s conference on institutional delivery, Northwest Ethiopia: comparative cross-sectional study. BMC Pregnancy Childbirth. (2019) 7:1–10. 10.1186/s12884-019-2537-7 PMC6790024 31606054

[29] Tekelab T Yadecha B Melka AS. Antenatal care and women’s decision making power as determinants of institutional delivery in rural area of Western Ethiopia. BMC Res Notes. (2015) 8:1–8. 10.1186/1756-0500-8-1 26651489 PMC4676818

[30] Envuladu EA Agbo HA Lassa S Kigbu JH Zoakah AI. Factors determining the choice of a place of delivery among pregnant women in Russia village of Jos North, Nigeria: achieving the MDGs 4 and 5. J Int Med Biomed Res. (2013) 2(1):23–7. 10.14194/ijmbr.215

[31] Gultie T Wasihun B Kondale M Balcha B. Home delivery and associated factors among reproductive age women. J Womens Health Care. (2016) 5(1):1–4. 10.4172/2167-0420.1000300

